# Stakeholders’ Perspectives on Implementing the iSupport for Dementia Program to Address Inequalities in Dementia Care: A Qualitative Study

**DOI:** 10.1111/scs.70255

**Published:** 2026-04-17

**Authors:** Lily Xiao, Kham Tran, Kate Laver, Claudia Meyer, Rachel Milte, Hui‐Chen (Rita) Chang, Ying Yu, Shahid Ullah, Anna Howard, Vicki Kanakaris, Mary Sophou, Barbara Leon, Alison Kitson

**Affiliations:** ^1^ College of Nursing and Health Sciences Flinders University Adelaide Australia; ^2^ South Adelaide Local Health Network Adelaide Australia; ^3^ Bolton Clarke Melbourne Australia; ^4^ School of Nursing and Midwifery Western Sydney University Sydney Australia; ^5^ Adelaide Nursing School The University of Adelaide Adelaide Australia; ^6^ College of Medicine and Public Health Flinders University Adelaide Australia; ^7^ Murray Mallee Aged Care Group Inc. Adelaide Australia; ^8^ Multicultural Aged Care Inc Adelaide Australia; ^9^ Family and Community Services Melbourne Australia; ^10^ United Melbourne Australia; ^11^ International Learning Collaborative Adelaide Australia

**Keywords:** community aged care services, cultural adaptation, culturally and linguistically diverse communities, dementia care, family caregivers, social inclusion

## Abstract

**Aims:**

To explore stakeholders' perspectives on (1) the cultural and linguistic appropriateness of the Greek, Italian and Spanish versions of the iSupport for Dementia program and (2) strategies to implement the iSupport program in Australia.

**Methods:**

A qualitative descriptive study was applied. Data were collected from focus groups with family carers of people with dementia from Greek‐, Italian‐ and Spanish‐speaking backgrounds and bilingual and bicultural health and social care professionals in community aged care settings in Australia. The reflective thematic analysis method was used to identify themes from the data. The COREQ checklist for qualitative research was followed to report this study.

**Results:**

We found that stakeholders would like to see inclusive language used in the iSupport program to empower and engage carers in the program. They also suggested that the iSupport program should meet diverse learning needs and preferences for carers. They would like to see bilingual and bicultural staff deliver the iSupport program to carers and coach carers based on their individual needs. They suggested that the methods used in carer peer support should be socially inclusive in the digital age. In addition, they would like to see the iSupport program integrated into aged care services.

**Conclusion:**

Implementing the iSupport for Dementia program in multiple languages that incorporates peer support and coaching activities for carers is an opportunity to address health inequalities for people with dementia and their carers from culturally and linguistically diverse backgrounds. Training and supporting bilingual and bicultural health and social care professionals is vital for embedding the iSupport program in aged care services.

## 1 Introduction

Training and supporting informal carers of people with dementia (PWD) has become a public health priority in countries with an aging population [1]. Home‐based care for PWD is more common in culturally and linguistically diverse (CALD) communities in high‐income countries than in mainstream cultures because of the influence of filial piety and collectivist culture [2]. Family carers from CALD backgrounds play a pivotal role in maintaining PWD at home [3]. However, they receive less support to achieve health and well‐being for PWD and themselves due to structural discrimination, which prevents them from accessing dementia care skill training and support [4, 5]. Health inequalities refer to differences in health and quality of life outcomes among populations due to their social status in a society [6]. We adapt this concept to the study context and describe health inequalities in dementia care as poor health and quality of life outcomes of PWD and their carers due to their CALD backgrounds. Contributing factors to health inequalities in dementia care are complex, ranging from the care recipient factors, carer factors and systemic factors [4, 7]. Health and social care professionals play a vital role in the cultural adaptation and implementation of evidence‐based dementia skills training and support programs to address health inequalities for PWD and their carers from CALD backgrounds in long‐term care. Thus, it is imperative to engage them and CALD carers of PWD in this study with a focus on factors affecting the implementation of the iSupport for Dementia program in real care settings to address inequalities in dementia care.

## 2 Background

Psychoeducation is a main type of non‐pharmacological intervention for carers and shows positive effects on improving carers' well‐being and self‐efficacy [8]. However, psychoeducation programs in CALD carers' preferred languages are lacking [2]. The WHO iSupport for Dementia program is an evidence‐based psychoeducation program developed to enable members' countries to achieve universal access to dementia care education for carers [9]. The program includes five modules described as: (1) introduction to dementia, (2) being a carer, (3) caring for me, (4) providing everyday care and (5) dealing with behaviour changes [9]. To date, over 40 countries have engaged in iSupport program adaptation and implementation [3, 10]. We received support from research teams in Greece, Spain and Switzerland to adapt their Greek, Spanish and Italian versions of the iSupport program to the Australian context [11, 12, 13]. However, consultations with relevant Australian stakeholders are imperative to ensure the appropriateness of the language and cultural experience within these translated versions and the best approach to implementing the program in these identified CALD communities.

Improving carers' mental health is the primary goal of psychoeducation interventions [8]. However, fully‐powered randomised controlled trial (RCT) using self‐guided iSupport intervention in the UK showed no effects on improving carers' mental health [10]. Such intervention results were attributed to a low‐level engagement of carers in the program, as evidenced by an average of four logins during a 6‐month intervention period. A similar finding was reported in the India iSupport trial, where the attrition rate in the intervention group was high (60.8%) [14]. These findings underscore the need to explore the barriers that CALD carers face when engaging them in the iSupport program.

Strategies that engaged carers in psychoeducation programs were explored in a recent review by Chan et al. [15]. First, training health and social care professionals to deliver psychoeducation programs to carers was viewed as a core component that motivated carers to learn and apply knowledge to their own care situations. Second, engaging carers in coaching activities to cope with the changed behaviours of their care recipients was found to be essential to reduce carers' mental stress. In addition, creating opportunities for carers to interact with their peers to share their experience in psychoeducation programs was a strategy to foster experiential learning while overcoming social isolation. A multicomponent intervention that incorporated carers' self‐directed learning using the iSupport manual, facilitator‐enabled carer peer support and access to care services demonstrated effects on carers' mental‐health‐related quality of life and self‐efficacy in controlling negative thoughts and distress reactions to changed behaviours of people with dementia in two RCTs in Australia and greater China [16, 17].

In Australia, rights‐based care underpins dementia and aged care policies [18], yet care services were viewed as better fitting carers from the mainstream culture group than those from the CALD group [5, 7]. To address the care needs of older people in the CALD group, ethno‐specific care organisations have been established [19]. These organisations usually employ bilingual and bicultural staff to meet older people's care needs relating to their culture and language use. However, these organisations have a limited capacity to provide psychoeducation programs to CALD carers. Dementia Australia is funded to provide education for carers; however, their programs are usually delivered in English [20]. The inequalities in dementia skills training programs and support for CALD carers are well‐recognised and viewed as a priority issue to be addressed in the National Dementia Action Plan [21].

## 3 The Study

The aims of the study were to explore stakeholders' perspectives on (1) the cultural and linguistic appropriateness of the Greek, Italian and Spanish versions of the iSupport for Dementia program and (2) strategies to implement the iSupport program in Australia.

## 4 Method

### 4.1 Design

We employed a qualitative descriptive design as explained by Doyle et al. [22]. This design enabled us to gather data in iterative rounds of consultations with stakeholders and describe their thoughts and ideas regarding the development and implementation of the iSupport program in the three languages. We applied the COREQ checklist for qualitative research to report this study [23].

### 4.2 Theoretical Framework

The ecological validity framework was applied to this study [24]. This framework underscores two levels of program adaptation in CALD populations: (1) the appropriateness of languages and contents in the program and (2) strategies to pragmatically implement the program in ethno‐specific care settings. The first level emphasises the appropriateness of the translation accuracy of the iSupport program and any further contents deemed beneficial to enhance psychoeducation for carers. The second level focuses on factors that may affect program implementation in community‐based ethno‐specific care settings.

### 4.3 Study Setting and Participants

Participants were purposively selected from Greek (*n* = 2), Italian (*n* = 1) and Latin American (*n* = 1) ethno‐specific care organisations across two Australian States. We developed an information sheet for carers and staff to explain the study and invite them to participate in the study. The participating organisations helped our researchers distribute the information sheet to potential carer and staff participants through their usual communication channels including newsletters, letters and emails. Potential participants who were interested in the study were directed to contact a site‐specific researcher for checking their eligibility to participate, discussing any questions they might have and signing a written informed consent.

### 4.4 Inclusion and Exclusion Criteria

Inclusion criteria for carer participants were: (1) aged 18 years or above; (2) providing care for an older person with dementia or probable dementia at least twice a week and (3) spoke Greek, Italian or Spanish at home. Carers with health conditions that could significantly impact their ability to participate in the study were excluded. The inclusion criteria for health and social care professionals were: (1) they could speak, read and write Greek, Italian or Spanish and (2) they had experience in caring for people with dementia and/or supporting their carers for at least 3 years. Staff members who were not direct care workers were excluded from the study.

### 4.5 Data Collection

We applied iterative rounds of stakeholder consultations as detailed in the following to collect data. The data collection methods enabled us to collect different types of data (data triangulation including review comments and focus group data) from different participant categories (participant triangulation including carers and staff). It also enabled us to have prolonged engagements with participants by engaging them in reviewing iSupport manuals, participating in focus groups, reviewing iSupport manuals again and reviewing focus group transcripts. These methods not only enabled us to achieve credibility in a qualitative study [25, 26], but also co‐design with stakeholders to culturally adapt the three language versions of the iSupport program [27, 28]. First, we sent a hardcopy version of the iSupport program with 1–2 translated modules in their preferred language and sought their feedback. Second, we invited them to participate in an online or face‐to‐face focus group in a meeting room of participating organisations lasting 90–120 min to elaborate their views on the iSupport program using a semi‐structured discussion guide (see Table 1). Questions 1–4 focused on the language and the content, while questions 5–9 focused on implementation strategies based on the ecological validity framework. Two researchers (L.X. and K.T.) conducted each focus group, with one as a moderator and the other as an observer, to take field notes to assist in data analysis. We conducted one language group at a time, and we did not separate staff from carers because of the small number of participants from each language group in each participating organisation. The moderator explained the rules that enabled each member to have an equal opportunity to discuss their views during group discussions. As most participants in the focus groups were bilingual, we conducted the discussions in English. However, we also employed an interpreter for each focus group to encourage carers to choose their preferred language in the discussions. The focus group discussions were audio‐recorded. Third, we revised the three language versions of the iSupport program and returned them to participants to check and provide further feedback when appropriate. Participants were given opportunities to view a summary of the findings based on the data collected in the study.

**TABLE 1:** A semi‐structured discussion guide.

A discussion guide for carer participants
What ideas do you have for improving the iSupport manual? What changes would you like to see in the iSupport manual? What content can be added or taken out from iSupport manual? What topics should be covered in the iSupport manual? How often would you like to use the iSupport manual? On a weekly basis, what is feasible time for you to spend studying the iSupport manual? If we implement the iSupport program in the aged care organisation, would you like to participate in it? What would be facilitators for you to participate in the iSupport program? What would be hindrances for you to participate in the iSupport program?
A discussion guide for staff participants
What ideas do you have for improving the iSupport manual? What changes would you like to see in the iSupport manual? What content can be added or taken out from iSupport manual? What topics should be covered in the iSupport manual? What would be facilitators for carers to participate in the iSupport program? What would be hindrances for carers to participate in the iSupport program? If we implement the iSupport program in the aged care organisation, would you like to participate in the program provision? What would be facilitators for you to participate in the iSupport program provision? What would be hindrances for you to participate in the iSupport program provision?

### 4.6 Data Analysis

Audio‐recorded focus‐group data were transcribed verbatim by a bilingual and bicultural researcher in each of the three languages. We returned transcripts to participants and invited them to crosscheck the transcripts to ensure accuracy. Transcripts or part of transcripts in Greek, Italian and Spanish were translated into English for data analysis. We employed the reflective thematic analysis method described by Nowell and colleagues [26] via the six interrelated phases: (1) familiarising yourself with your data; (2) generating initial codes; (3) searching for themes; (4) reviewing themes; (5) defining and naming themes and (6) producing the report. We detailed the application of these analytical phases in our study in Table 2. The data analysis method emphasises study rigour, the alignment of themes (findings) with the qualitative data and the aims of the study. The first and second authors who led the focus group also led the data analysis through the six phases of data analysis. The research team members also participated in the data analysis as detailed in Table 2.

**TABLE 2:** The application of the six phases of the reflective thematic analysis.

Six phases	Applications to the study context
Familiarising yourself with your data	The first and the second author read all transcripts and participants' comments on the translated iSupport manuals separately and reported the main points of these documents to the research team in monthly meetings. The second author organised all re‐identifiable data in a secured drive in an order for the research team to crosscheck when needed
Generating initial codes	The first author coded all qualitative data using the open coding method. The codes were crosschecked by the second author against the data. Differences on the codes were resolved based on the original data, the study aims and team discussions
Searching for themes	The first authors grouped the codes based on the similarities, analysed the concepts of the grouped codes against the aims of the study and suggested preliminary themes. The deductive thematic analysis based on the ecological validity framework was employed. Excerpts from the data were used to support the preliminary themes
Reviewing themes	The research team were invited to crosscheck and review the preliminary themes and the excerpts and suggest further changes
Defining and naming themes	The first author further refined the preliminary themes based on the suggestions from the research team. Differences on the themes were discussed in team meetings and email communications
Producing the report	The final report with rich descriptions of the study context, themes and excerpts were crosschecked, reviewed and revised by the team until they reached agreement

### 4.7 Ethical Consideration

Ethical approval was obtained from the Clinical Human Research Ethics Committee at the South Adelaide Local Health Network (project number: 2023/HRE00296). We distributed information about the study and consent forms to potential carers and staff participants in one of the four languages based on their preferences: English, Greek, Italian and Spanish. We assured the participants that there was no obligation to participate or that they could withdraw at any stage of the consultation process without any impact on them. Maintaining confidentiality of personal information within the study reports was ensured.

### 4.8 Study Rigour and Reflexivity

We made efforts to achieve study rigour. First, our trained bilingual and bicultural researchers engaged participants in multiple rounds of consultations. This prolonged engagement enabled the development of a trust relationship with participants by which participants were willing to share their perspectives of the iSupport program with the researchers. Second, we recorded focus group discussions, transcribed verbatim and returned the transcripts to participants to check and revise based on their perspectives. These processes ensured the credibility of the study. Third, we selected the most relevant excerpts to support the themes and linked the excerpts with the source of information. These approaches fostered the dependability of the study. Fourth, we used data from multiple study sites with both carers and staff participants to support findings. These data triangulation methods improved the confirmability of the study. Fifth, we organised team meetings regularly to discuss the data analysis, findings and seek feedback from the team. Differences on findings were resolved through discussions.

## 5 Results

Twenty carers and 20 care workers participated in the study. Participants' demographic information is presented in Table 3. We identified five themes from data analysis: (1) inclusive language to empower and engage carers in the program; (2) addressing the varied carers' learning needs and preferences; (3) bilingual and bicultural staff to engage with and support carers in the program; (4) socially inclusive approaches to connect carers with peers in the digital age and (5) engaging carers to participate in the program via existing care services. The findings are presented in detail in the following sections. We use the letters G, I and S with numbers to indicate that the quotes are from Greek, Italian and Spanish focus groups, respectively.

**TABLE 3:** Participant demographic information.

Participants characteristics	Carer participants *n* = 20	Staff participants *n* = 20
Age: mean (SD)	68 (12.0)	53 (9.5)
Gender: *n* (%)		
Male	2 (10.5%)	4 (20.0%)
Female	18 (89.5%)	16 (80.0%)
Culture		
Italian	5	5
Greek	10	10
Spanish	5	5
Years in the current role: mean (SD):	5.7 (3.9)	6.25 (6.3)
Full time/part time employment	N/A	Full‐time = 6 Part‐time = 3 Casual = 11
Relationship with the care recipient	Children = 11 Spouse = 9	N/A
Occupational categories	N/A	Nurse = 1 Allied health professionals = 3 Social care professionals = 7 Home care support worker = 7 Volunteer = 2

### 5.1 Theme 1: Inclusive Language to Empower and Engage Carers in the Program

The Italian focus group participants commended the linguistically accurate translation of the Italian version of the iSupport program but suggested further revisions to ensure language used in the translated version could empower carers: ‘I do have concerns about some of the words used in Italian, which are actually quite strong and negative. …For example, it is someone who lives with dementia rather than someone who is overwhelmed by dementia’ (I1). They provided a list of words to be replaced in the manual: ‘In module 5, they used the word wandering, and I looked it up, and it is not a word that is used in Italian … In reality, it is about being lost, a word that is much more widely used’ (I1).

The Greek focus group participants were aware of some low literacy levels in older carers. Hence, they questioned the suitability of the language for these carers: ‘Most of older carers had primary school education or no formal education. We need to use words that are much more widely used by this group of carers’ (G2).

The Spanish speaking focus group pointed out the sub‐cultures in the same language group and the need to use inclusive language in the program (S1): ‘The only thing is that it has been written in Spanish from Spain, so some of the words are slightly different from those used in South America’. ‘I found if the language is very simple, it makes the manual very very easy to understand even if you are from different parts of South America’. They discussed these solutions in the second Spanish speaking focus group: ‘I wonder if we need to simply put maybe a slash in some of the terms and offer the two options. Explaining different ways suit our migrants here’ (S2). Discussions revealed stakeholders' expectations of a program with empowerment and inclusive language.

### 5.2 Theme 2: Addressing the Varied Carers' Learning Needs and Preferences

Carer participants across the three language groups discussed the reasons for using the iSupport manual as a carer from an Italian speaking group said: ‘The main motive for me to use it is the fact that it is all in one manual that I can access the information I need’ (I1). However, carers expected more from the iSupport manual as a carer from a Spanish speaking group said: ‘I was wondering if the manual is going to present cases with a little bit more advanced dementia’ (S1). Carers from a Greek speaking group particularly mentioned: ‘There is a lot of loss and a lot of grief that need to be included’. ‘I would like to see things like having powers of attorney, having a will, ensuring that the family agrees’ (G1).

Staff participants pointed out the need to enable carers to learn to accept formal care services to reduce their stress as those from a Greek speaking group said (G3): ‘Many of our carers had illnesses. They are of a similar age to the person they care for’. ‘They are also reluctant to receive services because of lack of access to this information, but also the attitude of allowing an outsider to come into the home to provide care for their loved one’.

Carer participants across the three language groups would like to see more case studies in an Australian context to complement the iSupport manual as a carer from the Greek speaking group said: ‘I do have some younger carers who were born here in Australia and Greek is not the first language … So, adding case studies is needed to make the program more relevant to their experiences’ (G3). Older carers with low literacy levels preferred short videos to meet their learning needs: ‘I would like to go to videos that are contextualised in real situations. Each video should take approximately 5 min’ (S2). ‘Videos can help carers who cannot read or have limited reading abilities’ (I1). ‘Videos would be helpful because they are quick. You do not have to sort the read too much’ (G2). It was evident that carers expected the iSupport program to promote social inclusion to meet carers' learning needs and preferences in the program.

### 5.3 Theme 3: Bilingual and Bicultural Staff to Engage With and Support Carers in the Program

Carer participants strongly suggested that bilingual and bicultural staff trusted by carers were in an ideal position to deliver the iSupport program to them: ‘I think my motive in the program will be that you are encouraged by your community to read and inform yourself on how to look after people with dementia’ (S1). They used what they had experienced in participating in the iSupport consultations to reinforce their view: ‘I feel a sense of encouragement and support through Anthony [a staff who supported the carer to participate in the consultation] in the program consultation’ (G1).

Carers also expected a trained staff to coach them in dealing with difficult situations: ‘You know my husband has started smoking. It is not something I can manage. The doctor is also not able to manage it … I will benefit from someone giving me guidance and then I can grow to manage the situation’ (G1). The need for one‐on‐one coaching provided by a staff was reinforced by another carer: ‘My wife has hallucinations. I have those issues going on, so maybe more specific instructions by staff in the program would be helpful’ (I1).

Staff emphasised the trained staff's role in emotional support for carers: ‘The staff could help carers by discussing their feelings and encouraging them to perform self‐care’ (G2). Staff also discussed the support they needed from the iSupport program if they facilitated carers in the program: ‘I need a guide on how to do dementia education, refer carers to, and collaborate with other services in a way that more carers can benefit from’ (G3). Training needs for staff were reinforced by a staff participant: ‘So, we need to provide resources and training in the aged care sector. So, what you hear from me is training, training, education, education’ (I1).

### 5.4 Theme 4: Socially Inclusive Approaches to Connect Carers With Peers in the Digital Age

Staff from Greek speaking group were concerned about the social isolation that carers experienced and pointed out some factors that needed to be addressed in the program: ‘The carers I work mostly are in their 80s. They are very busy looking after their husband or wife and are very isolated’ (G1). ‘However, they don't drive. They are not very mobile, and most do not have access to technologies such as the Internet, iPads, and computers. Therefore, they have relied on landlines’ (G2).

Carers desired to communicate with peers in the iSupport program: ‘You want to talk to somebody that is going through the same circumstances that you are going through’ (S1). However, older carers had a limited option to connect with peers: ‘I look after my husband. I don't drive. It is via telephone, I can participate in any sort of future involvement with the program, but I cannot attend face‐to‐face meetings’ (G3).

Participants also suggested that peer support, respite care and trained staff‐led sessions should be combined when appropriate as discussed by participants from an Italian speaking focus group (I1):

Member 1: Many of them do not know how to use computers. Some of them have family members to help them use computers and iPads, but some of them do not have family members. Therefore, I think we will end up with group training for the entire manual.

Member 2: You turn the training more into social outings, which is actually good for both the carer and the person with dementia.

Member 3: That is what I am saying, but this is the ideal situation to go through the modules with a professional, with a support group in a lovely setting.

Discussions on flexible methods to accommodate carers' needs to connect and share their experiences with their peers in the program reinforced the need to foster social inclusion in carer peer support activities in the digital age.

### 5.5 Theme 5: Engaging Carers to Participate in the Program via Existing Care Services

Participants identified that the most appropriate way to engage carers to participate in the program was to embed the program in routine care services, which had implications for funding arrangements and staff capacity: ‘I think the program should be included in our service provision and ongoing. I guess it is a matter of funding and capacity of the organisation to have workers on board to support carers in the program’ (G1). Care participants would like the program to be funded via respite care services: ‘Perhaps we need to be given a bit more respite services to study the program’ (S2). Staff reinforced the need to relieve carers from their carers' duties to participate in the program via respite care services as participants from a Greek speaking focus group discussed (G1):

Member 1: Some carers cannot go out for an hour because they cannot leave the persons at home.

Member 2: The other way I can see is if there is assistance for the carer to have someone stay in their houses to look after the people they care for. In addition, the carers need to be given the option to come to our center to go through this manual and do it like a course for them and meet their peers. Transport support is needed for them.

Carer participants suggested the involvement of support workers in the program delivery: ‘The support workers who visit us can introduce the iSupport manual to the family’ (S2). Staff participants believed that their involvement in the iSupport program provision was an opportunity to collaborate with carers to improve care for their clients: ‘For me it is to gain feedback from the family about what's happening for the client and how to work together with the family around that’ (S1).

## 6 Discussion

Our study found that language promotes carers' positive thoughts and perceptions of their care roles. Previous studies have revealed the coexistence of carers' positive and negative thoughts [2, 29]. Filial piety, collectivist culture, spirituality and religiosity held by carers from CALD groups contribute to their positive thoughts about caregiving [2, 30]. Our study adds a new understanding to this research field that using language that is easy to understand and inclusive encourages carers to reframe their positive thoughts about caregiving. A systematic review confirmed that positive aspects of caregiving contribute to carers' mental wellbeing, quality of life, and self‐efficacy [31].

Our finding on carers' desire to learn how to care for a relative with late‐stage dementia supports previous studies that CALD carers cared for a larger proportion of people with dementia at home due to the underutilisation of residential care services [7, 32]. Our findings add evidence that carers are concerned about their role in the care of people in the late stages of dementia. Our findings on carers' desire to learn more about dementia care through case studies support other studies that reported carers' diverse experiences and learning needs [15, 30]. Carers' expectations for real case studies and videos to simulate case studies underscore the need to work with stakeholders to collect real cases, redevelop and present cases to engage carers in psychoeducation [15].

In our study, carers' desire to work with bilingual and bicultural staff in the iSupport program aligns with a previous study that identified facilitators' role in enabling carers to access resources and care services at the time of dementia diagnosis, coping with changed behaviours and managing the transition between care settings and types [33]. These aspects of support are vital for CALD carers, considering the widely reported hindrances to accessing and utilising formal care services due to language barriers and the lack of social networks [32, 34]. Moreover, systematic reviews confirm that self‐guided psychoeducation interventions show limited value in improving carers' mental health compared with those with a facilitator [35, 36]. Our findings on carers' expectations for the facilitator to coach them in managing the changed behaviours of their care recipients are in line with a recent review that confirms coaching as a strategy for carers to achieve their care goals through interactions with the facilitator [15].

Loneliness and social isolation are highly prevalent in the dementia carer population and contribute to poor social well‐being [37]. These situations are worse in CALD carers because of the limited number of carer support groups provided in their preferred language [5]. Virtual carer support groups using digital devices have been widely used to mitigate these social issues and have a positive impact on carers' social well‐being and mental health [38]. Participants in our study viewed the iSupport program as an opportunity for carers to connect with their peers to overcome social isolation. However, they were cautious about using virtual carer peer support because of the low digital literacy levels in some older carers. Our findings underscore the need to develop tailored support for CALD carers to connect with their peers and prevent inequalities in social support for carers in the digital age.

Our study supports previous studies that ethno‐specific care organisations are in an ideal position to reach hard‐to‐reach older people and their carers because of established trust relationships with CALD communities [19, 39]. However, we found that staff were concerned about funding to enable this delivery model. The aged care sector is viewed as a resource‐poor setting [40]. The staff viewed the iSupport program as an opportunity to collaborate with carers to improve dementia care. To this end, policy interventions are required to address funding issues in ethno‐specific and multicultural care services.

### 6.1 Strengths and Limitations of the Study

Our study demonstrated methodological strengths using the reflective thematic analysis method described by Nowell and colleagues [26]. This methodology enabled the research team to demonstrate data collection triangulation (review comments and focus groups), participant triangulation (CALD carers and staff from three language groups), and researcher triangulation in data analysis and interpretation of results (13 members with various expertise). These triangulations in a qualitative study enhance study credibility [25, 26]. Moreover, the use of the ecological validity framework [24] enabled the team to discuss strategies to implement the iSupport program with stakeholders in real care settings in ethno‐specific care organisations to ensure the iSupport program meets their expectations for dementia care services.

Our study has limitations. It only involved four well‐established ethno‐specific care organisations. The findings may not represent those in other CALD communities in Australia and countries similar to the Australian aged care context.

### 6.2 Recommendations for Further Research

Future research needs to explore the education preparation for bilingual and bicultural health and social care professionals to deliver an evidence‐based iSupport program that incorporates peer support and coaching activities for CALD carers. Moreover, future research should explore various peer support methods to meet CALD carers' needs to connect and share experiences with their peers. Furthermore, as suggested by stakeholders, culturally and linguistically appropriate case studies and videos will be developed to complement the iSupport program. In addition, strategies to implement the iSupport program suggested by stakeholders will be tested in our implementation study phase to improve the reach of the program to CALD carers.

### 6.3 Implications for Policy and Practice

First, the iSupport program needs to incorporate trained bilingual and bicultural staff to facilitate peer support and coaching for carers based on their need to enhance psychoeducation and social support for CALD carers. Second, training bilingual and bicultural care workers to implement the iSupport program is vital for embedding the program in existing care services. Third, policy intervention is vital to enable the iSupport program to be sustained in ethno‐specific care services to address existing inequalities in skills training and support programs for CALD carers.

## 7 Conclusion

Our study revealed that implementing an evidence‐based iSupport program in ethno‐specific aged care organisations requires training for bilingual and bicultural staff to facilitate carer peer support and coach carers in dementia care based on their individual needs. However, implementing such an iSupport program requires policy intervention to fund the program and train the staff to deliver the program. Researchers need to engage policymakers, ethno‐specific care organisations, carers and health and social care professionals to facilitate evidence‐informed policy and dementia care services to address health inequalities in dementia care using various strategies. For example, researchers will need to actively participate in dementia care policy reviews and revisions, engage key stakeholders throughout the research project by inviting them to the project advisory group, and involving them in reviewing research reports and implementation documents. Most importantly, researchers will need to make a great effort to incorporate evidence‐based care and support for PWD and their carers from CALD backgrounds in dementia care guidelines, procedures and clinical pathways.

## Author Contributions

Conceptualization: Lily Xiao, Kham Tran, Kate Laver, Claudia Meyer, Rachel Milte, Hui‐Chen (Rita) Chang, Ying Yu, Shahid Ullah and Alison Kitson. Data collection: Lily Xiao, Kham Tran, Anna Howard, Vicki Kanakaris, Mary Sophou and Barbara Leon. Data analysis: Lily Xiao and Kham Tran. Writing – original draft: Lily Xiao. Critical review and editing: Lily Xiao, Kham Tran, Kate Laver, Claudia Meyer, Rachel Milte, Hui‐Chen (Rita) Chang, Ying Yu, Shahid Ullah, Anna Howard, Vicki Kanakaris, Mary Sophou, Barbara Leon and Alison Kitson.

## Funding

This project is funded by the National Health and Medical Research Council (NHMRC) under the Targeted Call Research: Cultural, ethnic and linguistic diversity in dementia research 2022 (2024551).

## Ethics Statement

Ethical approval was obtained from the Clinical Human Research Ethics Committee at the South Adelaide Local Health Network (project number: 2023/HRE00296).

## Consent

We gained written consent from participants to participate in the study.

## Conflicts of Interest

The authors declare no conflicts of interest.

## Data Availability

The data that support the findings of this study are available by email lily.xiao@flinders.edu.au upon reasonable request.
